# Nicotine Upregulates the Level of Mcl-1 through STAT3 in H1299 Cells

**DOI:** 10.7150/jca.35453

**Published:** 2020-01-01

**Authors:** Maojun Zhou, Jinfeng Zhao, Qi Zhang, Xin Jin, Mingmei Liao, Lihua Zhang, Jiwei Wang, Manyi Yang

**Affiliations:** 1 Key Laboratory of Cancer Proteomics of Chinese Ministry of Health, Xiangya Hospital, Central South University, 87 Xiangya Road, Changsha, Hunan, 410008, People's Republic of China.; 2 Key Laboratory of Nanobiological Technology of Chinese Ministry of Health, Xiangya Hospital, Central South University, 87 Xiangya Road, Changsha, Hunan, 410008, People's Republic of China.; 3 Department of Hepatobiliary & Pancreatic Surgery, Xiangya Hospital, Central South University, Changsha, China.

**Keywords:** lung cancer, nicotine, Mcl-1, STAT3

## Abstract

**Background**: Nicotine contributes to development of human lung cancer and chemoresistance through activation of myeloid cell leukemia-1 (Mcl-1). Signal transducer and activator of transcription 3 (STAT3) generally participates in development and progression of human cancers. Therefore, we examined the STAT3 cascade in nicotine regulation of Mcl-1 transcription in human lung cancer cells.

**Methods**: The effects of nicotine on the expression of STAT3 and Mcl-1 were determined using western blot. The sub-cellular localization was tested using immunofluorescence. The activity of STAT3 promoter was checked using dual luciferase reporter assay.

**Results**: STAT3 was constitutively activated (i.e., tyrosine-phosphorylated, serine-phosphorylated and nuclear translocation), meanwhile the expression and transcriptional activity of Mcl-1 were up-regulated in lung cancer cells following treatment with nicotine. Transfection with siRNA targeting STAT3 or treatment with STAT3 inhibitor JSI-124 diminished Mcl-1 protein levels. Deleted mutagenesis of a putative STAT3 consensus binding sequence decreased Mcl-1 promoter activity and eliminated the increase of Mcl-1 promoter activity induced by nicotine. Abnormally, JAK (Jannus kinase) inhibitor AG490 can't induce the downregulation of Mcl-1 or inhibit the tyrosine-phosphorylation of STAT3. In addition, deactivated mutagenesis of STAT3 the tyrosine 705 site had no effect on the aggregation of STAT3 into nucleus induced by nicotine.

**Conclusions**: We have demonstrated that nicotine induces up-regulation of Mcl-1 through STAT3, which process may be independent on JAKs and not only dependent on the phosphorylation of Y705. Downregulation of Mcl-1 transcription by inhibiting STAT3 cascade may be a potential strategy for the treatment of this cancer.

## Introduction

Smoking is one of the most foreseeable causes of cancer, and has a positive correlation with mortality in 30% of cancer patients 1. Cigarette smoke contains more than 7000 compounds, at least 60 of which have been evaluated as carcinogens 2. Nicotine, the major component in tobacco, cannot initiate tumorigenesis in human 3. However, long-term nicotine exposure can induce the cell proliferation and epithelial-to-mesenchymal transition of lung cancer 4. Besides lung cancer, nicotine has also been evaluated in various tumors, including head and neck cancer 5, breast cancer 6, cervix cancer 7 and bladder cancer 8. Nicotine can enhance the survival of lung cancer cells and may contribute to development of human lung cancer and chemoresistance 9. However, the intracellular signal transduction mechanism remains enigmatic.

STAT3 (signal transducer and activator of transcription 3) is known as a transcriptional enhancer activated by IL-6 10. STAT3 transcriptional activity is activated by the phosphorylation of the tyrosine at 705 site, which can be directly catalyzed by nonreceptor tyrosine kinases such as Jannus kinase (JAKs) and receptor tyrosine kinase (RTKs) 10. When the residue tyrosin^705^ (Y705) in STAT3 is phosphorylated, the protein can form homo-dimers and translocate from cytoplasm to the nucleus, which is the canonical STAT3 pathway 11. Otherwise, the phosphorylation may occur at the residue serine^727^ (S727) in STAT3, which is required for maximal STAT3 activation 12. mTOR (mammalian target of rapamycin) and MAPK1 (mitogen-activated protein kinase 1) can catalyze the phosphorylation of STAT3 on S727 site^10^. Nicotine-induced chemoresistance is mediated by activation of STAT3 in bladder cancer cells 8, 13, in head and neck cancer cells 5, breast cancer cells 6, nasopharyngeal carcinoma 14 and lung cancer cells 15.

Myeloid cell leukemia-1 (Mcl-1), as a member of the Bcl-2 family, is considered as an antiapoptotic gene 16, 17. Over expression of Mcl-1 was found in various tumors 16. The 5'-flanking promoter region of Mcl-1 contains potential transcription factor binding sites with consensus sequences such as STAT, SRE, Ets, Sp1 and CRE-BP 18. Putative binding site for STAT3 was identified in the Mcl-1 promoter region 18. The transcription factor STAT3 has been confirmed to influence Mcl-1 expression. Activated STAT3 was shown to bind an SIE-related element of Mcl-1 promoter in large granular lymphocyte (LGL) leukemia 19. In cholangiocarcinoma, a STAT3 regulatory element was identified in the Mcl-1 promoter 20. The activation of STAT3 is promoted by B-RAF (V600E) activity and that the Mcl-1 promoter is dependent on a STAT consensus-site for B-RAF-mediated activation in melanomas 21. Otherwise, JAK2-STAT3-Mcl-1 signal transduction pathway existed in colorectal cancer 22, lung cancer 23, gastric cancer 24 and other solid cancers 25.

The present study was performed to determine whether Mcl-1 was mediated by STAT3 signal pathway induced by nicotine in human lung cancer cells. In H1299 cells, nicotine can induce the activation of STAT3 and expression of Mcl-1. Mcl-1 level is attenuated by STAT3 inhibitor JSI-124 and siRNA targeting STAT3. Mcl-1 promoter activity cannot be increased by nicotine when the STAT binding site is deleted. However, the JAK inhibitor AG490 has no effect on the expression of Mcl-1, and Y705 site may be not indispensable for activation of STAT3 following treatment with nicotine. In conclusion, in H1299 cells, nicotine induces the STAT3-Mcl-1 signal cascade, maybe independent of JAKs. The identified mechanism might provide basis for developing approaches to treatment of human lung cancer.

## Materials and methods

### Materials

Nicotine ((--)-Nicotine, Ditartrate - CAS 65-31-6) was purchased from Merk (Lot. No 481975). Inhibitor JSI-124 (Lot. No 238590) and AG490 (Lot. No 658401) were obtained from Calbiochem. Mcl-1 (D35A5) Rabbit (Lot. No 5453) and Stat3 (D3Z2G) Rabbit (Lot. No 12640) antibodies were from Cell Signalling Technology for western blot. Anti-STAT3 (phospho Y705) [EP2147Y] (Lot. No ab76315) and Rabbit monoclonal [E121-31] to STAT3 (phospho S727) (Lot. No ab32143) were purchased from Abcam for western blot. Alexa Fluor® 594 Goat Anti-Rabbit IgG (H+L) Antibody (Lot. No A-11012) and Alexa Fluor® 594 Donkey Anti-Mouse IgG (H+L) Antibody (Lot. No A-21203) were from Molecular Probes for immunofluorescence.

### Cell lines and culture conditions

The non-small cell lung cancer cells H1299 was purchased from Institute of Biochemistry and Cell Biology (Shanghai Institutes for Biological Sciences, CAS). H1299 was maintained in DMEM supplemented with 10% fetal bovine serum and cultured at 37 °C in a humid incubator with 5% CO_2_.

### Plasmids, siRNA and transfections

Mcl-1 promoter WT plasmid was provided by Haidan Liu (Department of Cardiothoracic Surgery, The Second Xiangya Hospital, Central South University, Changsha, Hunan). The Mcl-1 promoter mutant plasmid (the Mcl-1 promoter fragment without the STAT3 binding site) was constructed according to the QuikChange® Multi Site-Directed Mutangenesisi Kit (Stratagene). Y705W-STAT3 plasmid was a gift from Dr. Zhao (Second Military Medical University, Shanghai, China). These plasmids were transfected into H1299 cells using Lipofectamine 2000 (Invitrogen). For RNA interference, siRNA specific to STAT3 (5'-GGTACATCATGGGCTTTAT-3') was purchased from GenePharma Ltd. siRNAs were transfected into the cells using Lipofectamine RNAi max (Invitrogen) according to the instruction.

### Cell Extraction and Western Blotting

After treatment, the cells were harvested and the whole-cell extracts were prepared as the method previously described 26. Protein concentration was measured according to the BAC Assay Reagent. Western blot was performed as the protocol described before 26.

### Immunofluorescence

H1299 cells were seeded on coverslips in a 24-well plate. After transfected by at least 24h, cells were fixed and immofluorescence was done as previously described 26. After fixed for 15min in 4% formaldehyde solution, cells were washed three times in PBST (0.05% Tween-20 in PBS) and then blocked with 5% BSA in PBST at room temperature for 1h. Cells then were incubated with first antibody at 4 °C overnight. Following triple washing in PBST, cells were incubated with the second antibody for 1h at room temperature. After washing in PBS, cells were labeled with 0.2ug/ml DAPI in PBS for 5min, and then washed in PBS for three times. At last, cells were mounted in MOWIOL R4-88 reagent (Calbiochem) and photographed with a Carl Zeiss AxioVision 4 microscope.

### Transient transfection and dual luciferase reporter assay

H1299 cells were plated in 48-well plates and transfected with 500 ng/well DNA. The mixed DNA contained the indicated Mcl-1 promoter vector and 5ng/well of Rellina luciferase reporter plasmid (Promega), to allow normalization of data for transient transfection efficiency. After treated with nicotine, all of the samples were measured by a dual-luciferase reporter assay system (Promega). Luciferase activity was normalized to Rellina luciferase activity. Each experiment was performed at least in triplicate. Data were expressed as 'Luciferase activity' (mean±sd) relative to control (transfected with empty vector).

### Statistical analysis

The data were subject to statistical analysis using the SPSS software package (version 19.0). All data were expressed as the means ± SD of at least three independent experiments. The statistical differences were analyzed by the Student's t-test. The differences were considered to be statistically significant at P<0.05.

## Results

### Constitutive STAT3 activation is induced by nicotine

**Figure 1:**
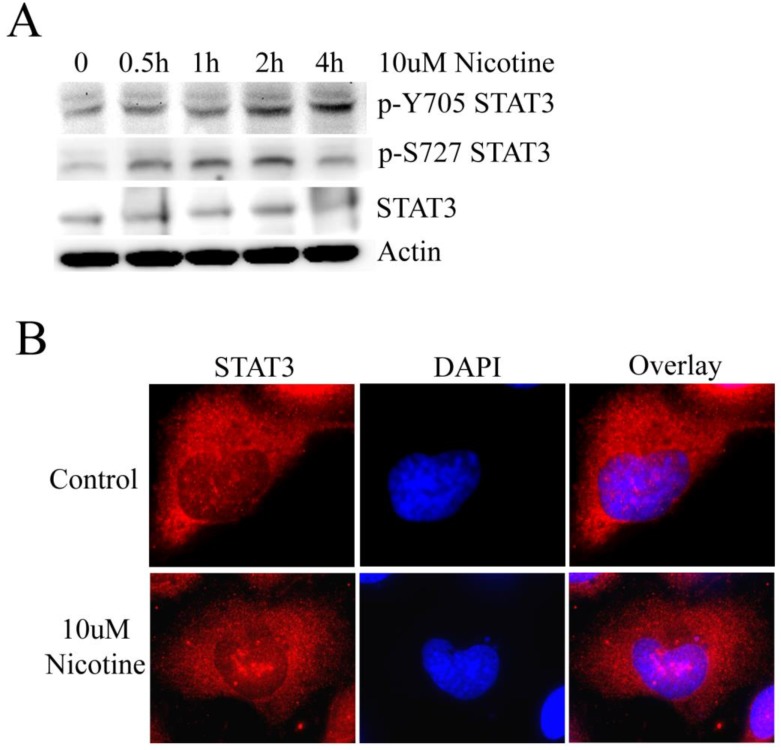
** Nicotine can induce the activation of STAT3. (A)** H1299 cells were treated with 10µM nicotine for 0h, 0.5h, 1h, 2h and 4h. Western blot was done to check p-Y705 STAT3, p-S727 STAT3 and STAT3. **(B)** 10µM nicotine treated H1299 cells for 2h and the the immunofluorescence was done to check the subcellular localization of STAT3. Untreated cells were done as control.

STAT3 has been shown to be stimulated by IL-6 in cholangiocarcinoma cell lines 20. However, whether STAT3 plays a role in lung cancer cells treated by nicotine remains to be clarified. To address this issue, we initially evaluated whether STAT3 was constitutively activated by nicotine in lung cancer cells. Usually, STAT3 activation is measured by the phosphorylation at Y705 and S727 site. Therefore, H1299 cell line was treated with 10µM nicotine for 0h, 0.5h, 1h, 2h and 4h respectively. The results of western blot show that the level of p-Y705 STAT3 increases following treatment with 10µM nicotine in a time-dependent manner; and the level of p-S727 STAT3 is markedly higher when treated with nicotine at 0.5h, 1h and 2h; but the expression of STAT3 changes little (Figure 1A). When phosphorylated at residue Y705, STAT3 undergoes translocation from the cytoplasm to the nucleus, where it functions as a pivotal transcription factor regulating gene transcription 10. So immunofluorescence was done to check the subcellular localization of STAT3 after cells were treated with or without nicotine. Actually, STAT3 diffusely localized at both nucleus and cytoplasm. After H1299 cells were treated with nicotine, much STAT3 translocated and aggregated at nucleus as bigger speckled formation (Figure 1B). These results led to the conclusion that STAT3 is constitutively activated in H1299 cells following treatment with nicotine.

### Nicotine increases the level of Mcl-1 and has an effect on the Mcl-1 promoter transcriptional activity

**Figure 2:**
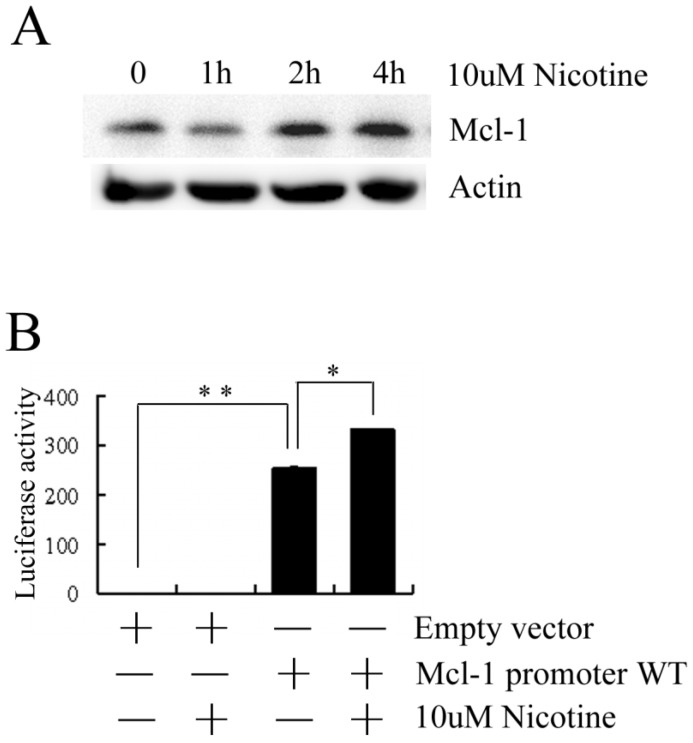
** Nicotine upregulates Mcl-1. (A)** H1299 cells were treated with 10µM nicotine for 0h, 1h, 2h and 4h. Western blot was done to check the expression of Mcl-1. **(B)** H1299 cells were transiently transfected with the luciferase reporter plasmid containing a 325bp long human Mcl-1 promoter fragment and Rellina luciferase plasmid and then treated with or without 10µM nicotine for 2h. Samples were measured by a dual-luciferase reporter assay system. Empty vector was transfected as control. Each experiment was performed at least in triplicate. Empty vector: original pGL3-Basic plasmid; Mcl-1 promoter WT: a 325bp long human Mcl-1 promoter fragment was inserted into pGL3-Basic plasmid. Columns represent the mean of three separate determinations; bars, SD; *P<0.05, **P<0.01(Student's t-test).

To investigate the expression pattern of Mcl-1 following treatment with nicotine, Mcl-1 expression was firstly measured by western blot. As shown in Figure 2A, the level of Mcl-1 is markedly higher when treated with nicotine at 2h and 4h. This result suggests that nicotine can induce the expression of Mcl-1. To investigate whether nicotine has an effect on the promoter of Mcl-1, H1299 cells were transiently transfected with the luciferase reporter plasmid containing a 325bp long human Mcl-1 promoter fragment and then treated with 10µM nicotine. As shown in Figure 2B, transfection of the Mcl-1 promoter WT plasmid generates much higher luciferase activity than that of the empty vector, which indicates the high transcriptional activity of Mcl-1 promoter in H1299 cells. And the luciferase activity of H1299 cells transfected with Mcl-1 promoter WT plasmid increased notably following treatment with nicotine, which implies the involvement of nicotine in the Mcl-1 promoter transcriptional activity.

### Nicotine-mediated STAT3 cascade regulates Mcl-1

**Figure 3:**
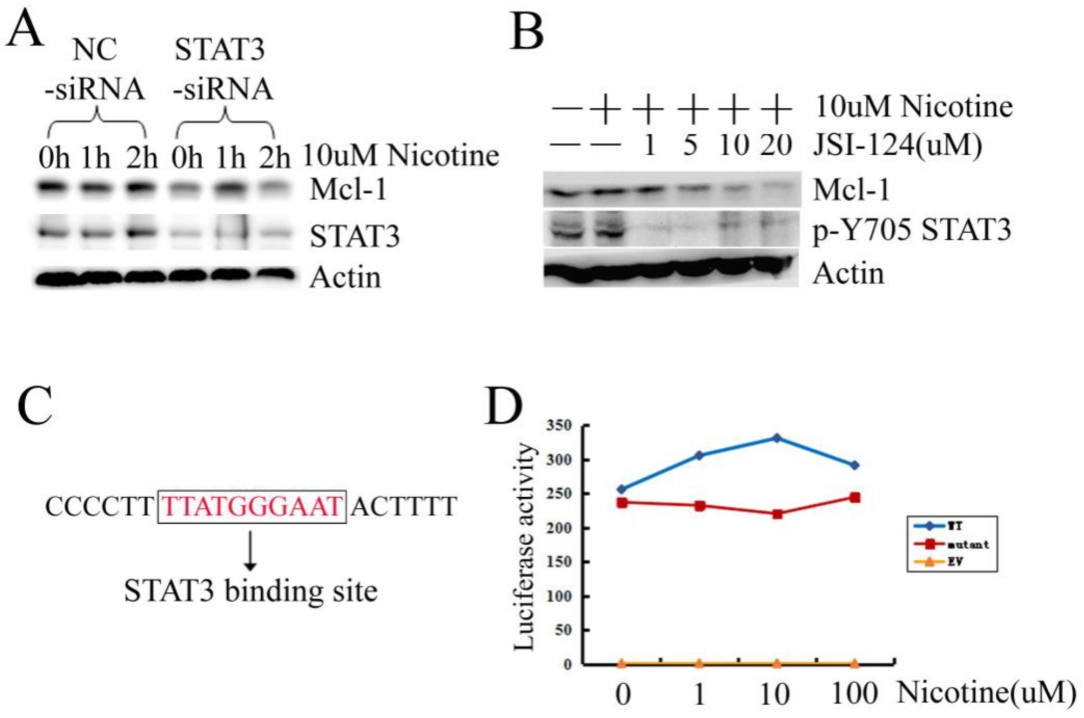
** Activated STAT3 induced by nicotine regulates Mcl-1. (A)** H1299 cells were transiently transfected with siRNA targeting STAT3 (STAT3-siRNA) and negative control siRNA (NC-siRNA) at least 24h, and then treated with 10µM nicotine for 0h, 1h and 2h. Western blot was done to check the expression of Mcl-1 and STAT3. **(B)** H1299 cells were treated in the absence or presence of increasing concentration of JSI-124 for 30min, and then treated with 10µM nicotine for 2h. Western blot analysis was performed to detect the p-Y705 STAT3 and the expression of Mcl-1. **(C)** The sequence of Mcl-1 promoter containing a STAT3 binding site. **(D)** H1299 cells were transiently transfected with EV, WT, mutant luciferase reporter plasmid and Rellina luciferase plasmid, then treated in the absence or presence of increasing concentration of nicotine for 2h. Samples were measured by a dual-luciferase reporter assay system. Each experiment was performed at least in triplicate. EV: original pGL3-Basic plasmid; WT: a 325bp long human Mcl-1 promoter fragment was inserted into pGL3-Basic plasmid; mutant: the Mcl-1 promoter fragment without the STAT3 binding site was inserted into pGL3-Basic plasmid.

We further confirmed whether STAT3 was involved in Mcl-1 expression in H1299 cells. Firstly, we designed the siRNA targeting STAT3. When STAT3 was silenced by siRNA, the expression of STAT3 and Mcl-1 were downregulated simultaneously (Figure 3A). Then inhibitor JSI-124, which suppresses STAT3 tyrosine phosphorylation, was used. Treatment of H1299 cells with JSI-124 resulted in a dose-dependent attenuation of Mcl-1 expression (Figure 3B). There is a putative consensus STAT3 binding site (TTATGGGAAT) at position -92/-83 within the promoter of Mcl-1 (Figure 3C). Then the mutant of Mcl-1 promoter, with the deletion of the STAT3 binding site, was constructed to luciferase reporter plasmid (named Mcl-1 promoter mutant). The luciferase activity assay displays that Mcl-1 promoter mutant loses the promoter activity a little (Figure 3D). However, when the STAT3 binding site was deleted in the Mcl-1 promoter, nicotine could not increase the luciferase activity as what it did in the Mcl-1 promoter WT transfected cells (Figure 3D). Taken together, these results provide evidence that STAT3 is involved in Mcl-1 expression in H1299 cells treated with nicotine via mediating the transcriptional process.

### Nicotine may stimulate STAT3 and upregulate Mcl-1 independent of JAKs and the phosphorylation of Y705 site is not indispensable

**Figure 4:**
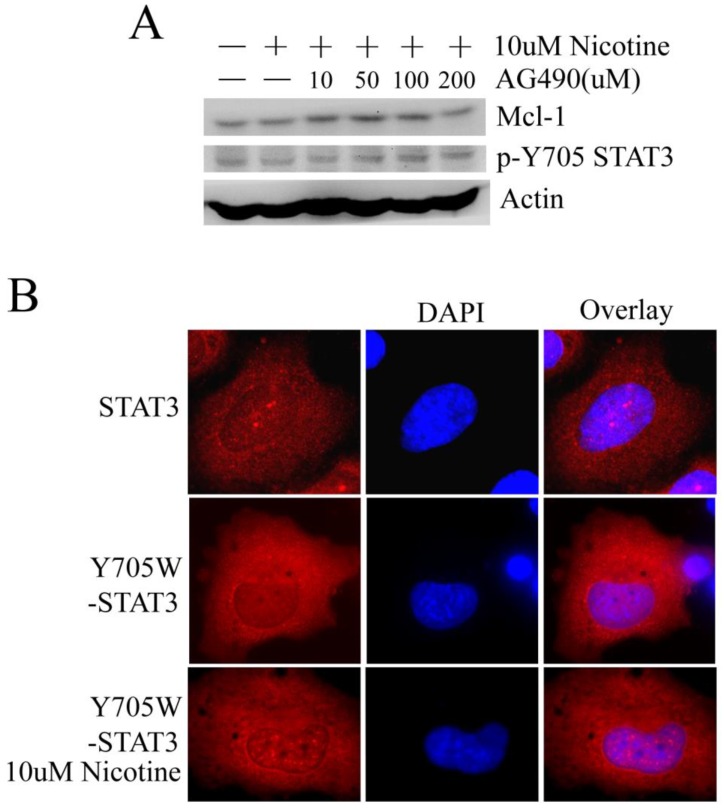
** Nicotine may stimulate STAT3 in a JAKs-independent manner and the phosphorylation at Y705 site isn't indispensable for nuclear accumulation of STAT3. (A)** H1299 cells were treated with 10µM nicotine in the absence or presence of increasing concentrations of AG490 for 2h. Phosphosylation of STAT3 and expression of Mcl-1 were determined by Western blot. **(B)** Immunofluorescence was done to check the subcellular localization of STAT3 using STAT3-antibody. H1299 cells were transiently transfected with Y705W-STAT3 plasmid (with Flag tag) at least 24h and then immunofluorescence was done with Flag-antibody. Y705W STAT3: the residue Y at 705 site of STAT3 was mutated to W.

Tyrosine phosphorylation of STAT3 can be directly catalyzed by non-receptor tyrosine kinases such as JAKs 10, which can be inhibited by AG490. However, in H1299 cells, AG490 cannot reduce the phosphorylation of STAT3 at Y705 site and the expression of Mcl-1 changed little following AG490 treatment (Figure 4A). Such result suggests that nicotine may activate STAT3 a JAKs-independent manner.

STAT3 activated by the phosphorylation of tyrosine residue Y705 is the canonical STAT3 pathway. When the residue Y mutated to W at 705 site (inactivated mutant), the immunofluorescence pictures show that nicotine still can induce STAT3 to translocate and aggregate at nucleus in H1299 cell line (Figure 4B). Thus, we suppose that nicotine may stimulate STAT3 not only dependent on phosphorylation at Y705 site.

## Discussion

Our previous study suggests that nicotine-prolong cell survival is closely associated with Mcl-1 in small cell lung cancer cells (H69 and H82) and non-small cell lung cancer cells (H1299 and H157) 9. Those findings reveal that Mcl-1, which is the major anti-apoptotic member of Bcl-2 family, may play a more extensive role in survival and chemoresistance of human lung cancer cells, especially in those cells that express low or undetectable levels of endogenous Bcl-2^9^. The previous study has demonstrated that nicotine-enhanced survival of lung cancer cells may occur through activation of Mcl-1 by phosphorylation. This study is in order to examine whether Mcl-1 is regulated at transcriptional level in human lung cancer cells induced by nicotine. H1299 cells are exactly the lung cancer cells that express high levels of endogenous Mcl-1 but do not express Bcl-2. And Mcl-1 may play more important roles in cells that express undetectable levels of Bcl-2. Therefore, in order to investigate the transcriptional mechanism of Mcl-1, H1299 cells were chosen.

Tyrosine 705 and serine 727 phosphorylations are required for STAT3 dimerization and maximal transactivation, respectively 27. Phosphorylation of the conserved Y705 residue can induce STAT3 to polymerize with itself or other STAT molecules, and then translocate to the nucleus, where it binds to specific DNA elements 10. Nicotine can induce the phosphorylation of Y705 and S727 residues of STAT3 and the aggregation of STAT3 in nucleus (Figure 1). However, when the Y705 site was mutated to W, nicotine still can mediate the aggregation of STAT3 into nucleus (Figure 4B). Maybe, besides phosphorylation, some other mechanisms that activate STAT3 following stimulation of nicotine exist in lung cancer cells. Usually, JAKs can induce the tyrosine phosphorylation of STAT3. Unexpectedly, JAKs inhibitor AG490 had little effect in our system (Figure 4A). Therefore, when H1299 cells were treated with nicotine, tyrosine phosphorylation of STAT3 may be catalyzed by receptor tyrosine kinase (RTKs) such as EGFR, KDR and MET or by other unknown mechanism.

Nicotine could activate the antiapoptotic function and enhance the half-life of Mcl-1, which led to its long-term survival function and chemoresistance of human lung cancer cells 9. The results in this study demonstrate that the expression level of Mcl-1 is up-regulated by nicotine (Figure 2A). So we supposed there should be a factor mediating transcription of Mcl-1. Mcl-1 expression can be increased by STAT3 pathway in cholangiocarcinoma cells 20, colorectal cancer cells 22, melanoma cells 28 and gastric cancer cells 24. As expected, when STAT3 was inhibited by siRNA or JSI-124, expression of Mcl-1 decreased obviously in H1299 cells (Figure 3A-B). When the STAT3 binding site in promoter of Mcl-1 was deleted, nicotine could not increase the transcriptional level of Mcl-1 in a dose-dependent manner (Figure 3D). However, the deleted mutant promoter of Mcl-1 without STAT3 binding site still has high level transcriptional activity, just lower than the wide-type promoter a little. Therefore, the STAT3 binding site may be not the only one that mediates transcriptional activity induced by nicotine in H1299 cells. According to the literature, in the 5'-flanking region of Mcl-1, there are several transcription factor binding sites including STAT, SRE, Ets, Sp1 and CRE-EP 18. So we are sure that other transcription factors may participate in up-regulating Mcl-1 induced by nicotine in H1299 cells.

Tobacco smoking has been reported to be associated with increased risk of cardiovascular disease and cancer, particularly of the lungs. Nicotine, the major addictive compound in cigarette smoke, is known to induce chemoresistance in some cancer cells. Our previous study has verified that nicotine can enhance survival of lung cancer cells through activation of Mcl-1 by phosphorylation 9. And the present study demonstrates that Mcl-1 is transcriptional regulated by STAT3 pathway induced by nicotine in human lung cancer cells. However, both of our studies just reveal the intra-cellular mechanism in human lung cancer cells following nicotine treatment, without any clinical data. Nowadays, we are collecting surgically resected human tumor samples. Next step, we will validate the potential mechanism revealed in the two studies in human tumor samples.

**Figure 5:**
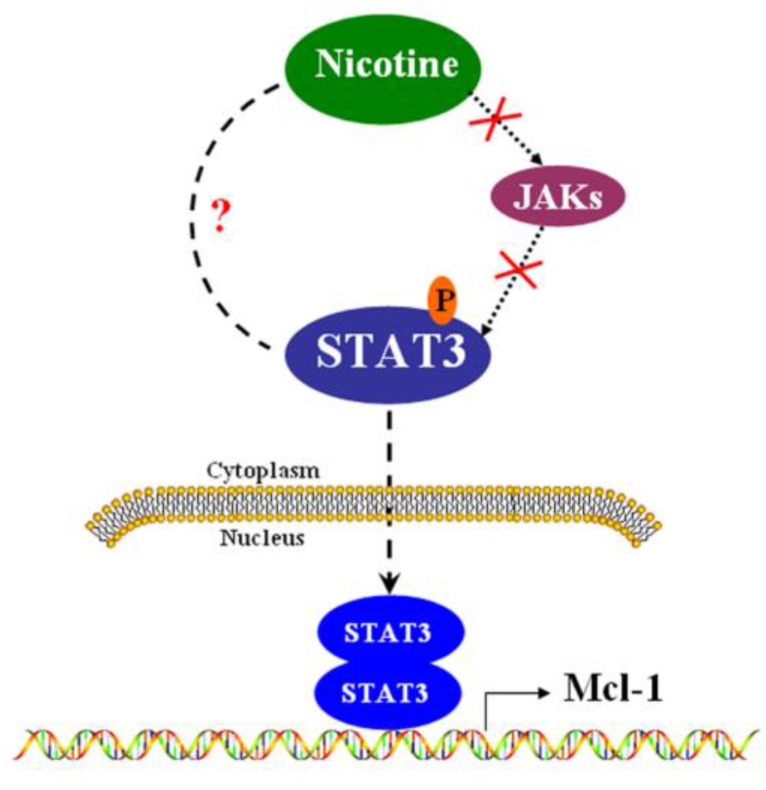
** The signal pathway that nicotine up-regulates Mcl-1 via activating STAT3 in H1299 cells.** In H1299 cells, nicotine induces the phosphorylation of STAT3 in a JAKs-independent manner. Phosphorylated STAT3 can translocate into nucleus, where STAT3 may form homodimers and regulate the promoter of Mcl-1.

In summary, we have provided evidence regarding how Mcl-1 is regulated at transcriptional level in H1299 cells (Figure 5). The present study identifies that STAT3 contributes to Mcl-1 production through STAT3 binding site within Mcl-1 promoter. In another word, Mcl-1 is the down-stream target of STAT3, but the canonical JAK is not the up-stream kinase of STAT3 in H1299 lung cancer cells. The newly identified mechanism suggests that targeting the STAT3 pathway might improve treatment results in some human lung cancer with high levels of Mcl-1.
